# Comparison of outcomes after total hip arthroplasty between patients with osteonecrosis of the femoral head in Association Research Circulation Osseous stage III and stage IV: a five-year follow-up study

**DOI:** 10.1186/s13018-024-04617-y

**Published:** 2024-03-06

**Authors:** Tianyu Wang, Dongwei Wu, Chengsi Li, Xinqun Cheng, Zhenbang Yang, Yingze Zhang, Yanbin Zhu

**Affiliations:** Department of Orthopaedic Surgery, The 3r, Hospital of Hebei Medical University, NO.139 Ziqiang Road, Shijiazhuang, 050051 Hebei People’s Republic of China

**Keywords:** Osteonecrosis of the femoral head, ARCO stage, Total hip arthroplasty, Outcomes

## Abstract

**Background:**

No large cohort study has evaluated the surgical outcomes of THA between different stages of ONFH patients. This study aimed to compare the surgical outcomes of ONFH patients who underwent THA in ARCO stage III versus IV, in terms of operative parameters, one-year hip function assessments and postoperative at least five-year complications, to inform optimized management of ONFH.

**Method:**

From our prospectively collected database, 876 patients undergoing THA between October 2014 and April 2017 were analyzed and divided into ARCO stage III group (*n* = 383) and ARCO stage IV group(*n* = 493). Details of demographics, medical record information, adverse events and clinical scores of both groups were collected and compared. Proper univariate analysis was used for the analysis.

**Result:**

There were no statistically significant differences in baseline characteristics between the two groups. Compared to ARCO stage IV patients, ARCO stage III patients showed a shorter operative time (*p* < 0.01), less bleeding (*p* < 0.01), fewer one-year readmissions (*p* = 0.026) and complications (*p* = 0.040), and significantly higher HHS (*p* < 0.01) one year after THA. In addition, ARCO stage IV patients seem more likely to suffer prosthesis dislocation (*p* = 0.031).

**Conclusion:**

Although ARCO stage IV patients in the study cohorts appeared to suffer more one-year complications, no significant difference was observed at long-term follow-up. Enhanced clinical guidance on preventing early prosthesis dislocation may help improve the prognosis of final-stage ONFH patients.

## Introduction

Osteonecrosis of the femoral head (ONFH) is a common painful and disabling orthopaedic disease, which is estimated to affect more than 20 million people worldwide [1–3]. The new released Association Research Circulation Osseous (ARCO) clinical guideline suggests that total hip arthroplasty (THA) is the most effective treatment for relieving pain and restoring mobility in ONFH patients whose femoral head has progressed to the collapsed stages [4–6]. However, substantial high-level evidence implicates that if THA is performed too early in young, active patients, it shows poor long-term outcomes, shorten implant survival time, and requires at least one and even multiple revisions [7]. Clinically, for those who are young or have not yet experienced secondary osteoarthritis, the surgeons tend to choose hip preservation treatment and delay the time of THA until the final stage of ONFH [8]. Unfortunately, this may increase the risk of postoperative adverse events of THA and compromise its clinical efficacy.

Several studies have shown that most of osteonecrosis and hip deformity steadily worsened with time [9, 10]. According to the ARCO staging system, ARCO stage III ONFH patients shows only subchondral fractures and surface collapse. But as time goes on, the hips appears osteoarthritis, acetabular destruction, and joint space narrowing, which means the disease has advanced to ARCO stage IV [4]. To our knowledge, few existing studies have explored the differences in outcomes of THA performed at different ARCO stages, and sporadically published ones are also limited by inadequate design, insufficient study variables and small sample size. For example, Jo et al. showed that delaying THA to advanced stage reduced postoperative hip motion in a controlled study of ONFH patients after THA at different ARCO stages [8]. However, they focused only on hip function and ignored other outcomes such as intraoperative information and postoperative adverse events. Through the review of ONFH subjects in different ARCO stages, Yang et al. suggested that ARCO stage III patients obtained less operative trauma and better functional recovery than ARCO stage IV patients [11]. Unfortunately, their study group is small, consisting of over a hundred people. In addition, their follow-up time was only one year.

In recent years, with growing population of ONFH patients and its younger trend, more young patients will be challenged by the proper time of THA [12, 13]. The aim of this study was to compare the THA outcomes of ONFH patients in ARCO stage III versus IV, focusing on operative parameters, one-year hip function assessments and postoperative at least five-year complications, to inform optimized management of ONFH.

## Materials and methods

### Data source

All data for this cross-sectional study were derived from the Surgical Site Infection in Orthopedic Surgery (SSIOS) database, which is a prospectively manually maintained database of all data on hospitalized patients who underwent orthopedic surgeries in our institution, beginning from 1 October 2014 and updated annually. Our institution is a teaching hospital and the largest tertiary referral medical center for orthopedic trauma in a China’s central province (with over 75.9 million population of the catchment), in which 45,000–50,000 orthopedic surgeries are performed annually. The data are collected manually by 230 standardized trained investigators and updated annually; the database has provided data support in many previous studies [14–17]. The information is more accurate than in the administration databases due to manual collection and update. Therefore, the data analyzed in this study can be considered of high quality and precision.

### Study design and participants

We prospectively collected hospitalised patients aged 18 years or older who underwent primary unilateral THA for ONFH at the Third Hospital of Hebei Medical University from October 2014 to April 2017. Patients were not considered in the study when: (1) incomplete study data; (2) conversion surgery after other hip surgery; (3) bilateral hip arthroplasty; (4) revision total hip arthroplasty; (5) symptoms of other large joint lesions; (6) serious systemic illness; (7) psychiatric disorders; (8) lost of follow-up. This was done for two reasons: (1) most THAs are primary unilateral THAs, of more clinical interest; and (2) to keep the good population homogeneous and allow easy interpretation of results. This study was approved by the Ethics Committee of the Third Hospital of Hebei Medical University. All procedures were performed according to the principles of the Declaration of Helsinki and in accordance with the guidelines of Strengthening the Reporting of Surgical Cohort Studies (STROCSS). Informed consent was obtained from all patients involved in this study. And to protect the privacy of patients, all data were anonymized by removing sensitive personal information.

### Data collection

Patient demographic information included age, sex (male or female), bad addiction (smoking or drinking), chronic disease (hypertension or diabetes), residence (rural or urban), height, and weight. BMI was calculated from height in meters and weight in kilograms. The preoperative diagnosis was recorded and the stage of ONFH was determined by imaging. The ONFH stage depends on the Association Research Circulation Osseous (ARCO) staging system. We use the ASA index to assess the patient's physical condition and surgical risk. Surgical information collected included the side of surgery, the anesthesia method, the duration of surgery, intraoperative bleeding, and intraoperative blood transfusion. The duration of surgery was calculated from the time of the skin incision to the time of the skin closure. Length of stay (LOS) was also collected and recorded, which we defined as the number of days from the date of admission to the date of discharge.

All complications and revision that occurred during the 5-year follow-up period were recorded. Local complications are those occurring at the level of the surgical site, and included pain, superficial infection, deep infection, hematoma, periprosthetic fracture and dislocation. Systemic complications included anemia, deep vein thrombosis, pulmonary complications, gastrointestinal, neurological, cardiovascular and genitourinary complications. The readmission and mortality were defined as any unplanned readmission and death case clearly associated with THA to any hospital within one year. When multiple adverse events occurred, only the first time was included in the analysis. The Harris hip score (HHS) for hip function and visual analogue scale (VAS) scores for pain were measured separately before surgery and 12 months after surgery [18, 19].

### Surgical procedures

The procedures were performed by a posterolateral approach and combined with spinal-epidural analgesia or general anesthesia, as determined by the anesthesiologist on the side. A curved incision of approximately 10 cm was made through the posterior and lateral approaches to the hip joint, and the skin, subcutaneous tissue, and fascia were incised in sequence. The lower limb was straightened and rotated inwards to expose the stop of the external rotator muscle group behind the greater trochanter and the joint capsule inside. The joint capsule was incised, and the hip was then dislocated by inward tucking and internal rotation. The femoral head was amputated approximately 1.5 cm above the lesser trochanter. The joint capsule and its surrounding synovial tissue were removed along the acetabular rim, and the acetabulum was gradually expanded and deepened. After the acetabular fitting successfully, a prosthesis was placed. The affected limb was kept in knee flexion, hip flexion and internal rotation. The femoral stem prosthesis and femoral head prosthesis were installed, and the joint was repositioned. The trauma cavity was irrigated and cleaned, and the incision was sutured layer by layer.

### Patient management

A strict perioperative strategy was implemented when patients were admitted to the hospital. All procedures were performed by surgeons with specialized training in a single institution. Cementless prostheses and ceramic-on-ceramic bearing were used in all patients. All patients maintained the affected limb in an abducted neutral position after THA, and their limbs were fixed with an anti-rotation shoe. Postoperative antibiotics were administered prophylactically. Patients were encouraged to early move down to the floor and carry out activities, including walking with crutches, partial weight-bearing, and strengthening exercises for abductor muscles. Medical staff instructed patients to review at 1, 3, 6, and 12 months after discharge, then follow-up visits by telephone every two years.

### Statistical analysis

Based on the preliminary study, enrolled patients were required for statistical analysis. All statistical analyses were conducted with the Statistical Package for Social Sciences (SPSS) software (version 26.0, Chicago, USA). Categorical variables were presented as numbers with percentages. For comparison, the Pearson Chi-square test was used, and if group counts were < 5 the Fisher Exact test was applied. Continuous variables are presented as the mean and standard deviation. If normally distributed, the Student's t-test was applied to compare means. If not, the nonparametric Mann–Whitney U test was used to compare medians. Significant differences were indicated by *p* < 0.05. 60-month complication-free rate was estimated with the Kaplan–Meier method.

## Result

### General information

**Fig. 1:**
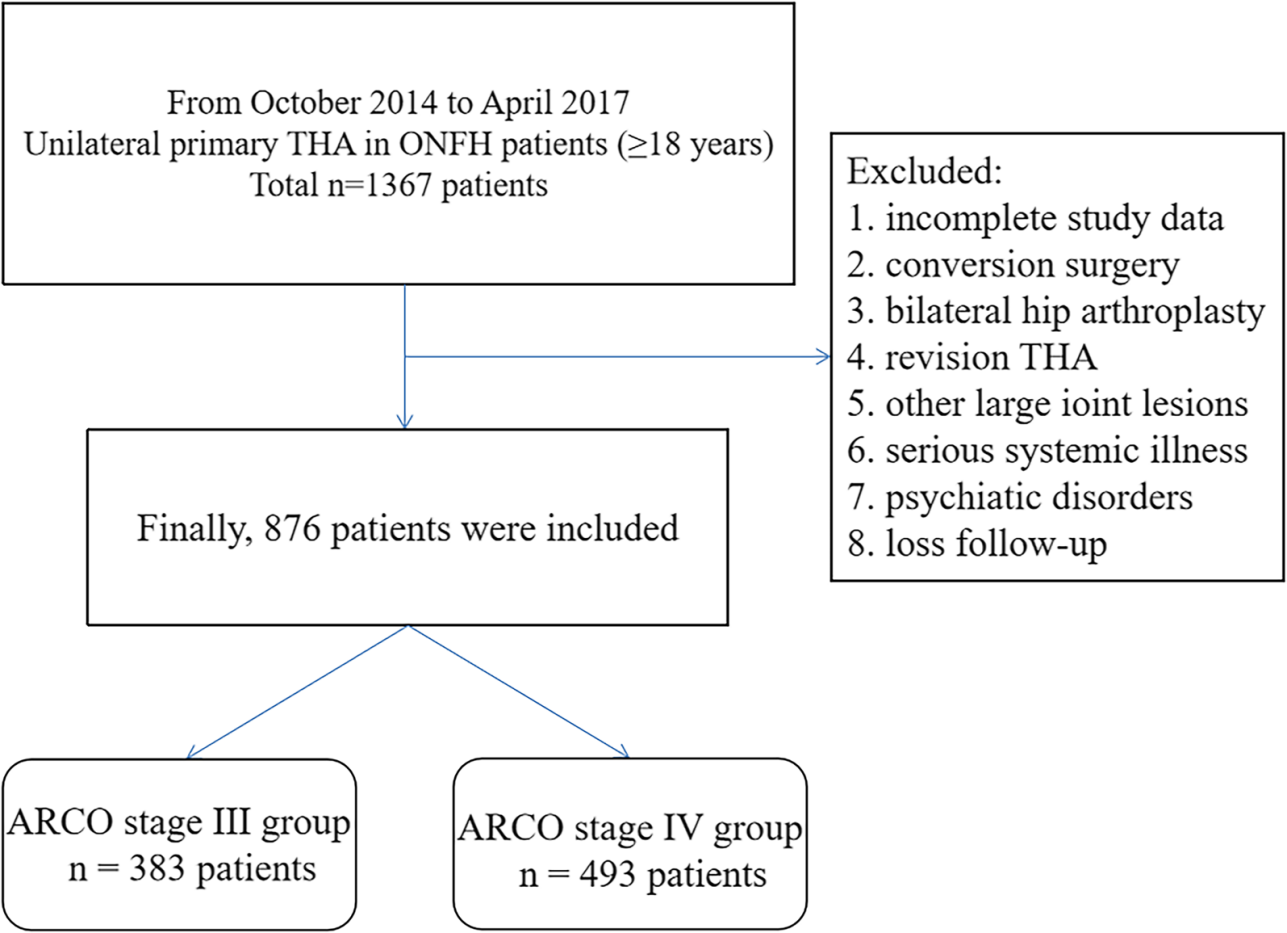
Sample selection flow chart

Finally, a total of 876 ONFH patients who met the eligibility criteria were enrolled and analyzed in this study, including 383 patients in ARCO stage III (group A) and 493 patients in ARCO stage IV (group B) (Fig. 1). Table 1 summarized the baseline characteristics of the two groups. There were no statistically significant differences in age (*p* = 0.687), sex (*p* = 0.567), BMI (*p* = 0.976), place of residence (*p* = 0.825), smoking (*p* = 0.579), alcohol use (*p* = 0.331), hormone use (*p* = 0.285), hip injury experience (*p* = 0.262), painkiller (*p* = 0.645), hypertension (*p* = 0.333), diabetes (*p* = 0.701), anemia (*p* = 0.868), heart disease (*p* = 0.700), cerebrovascular disease (*p* = 0.169), pulmonary disease (*p* = 0.465), liver disease (*p* = 0.102), urinary system disease (*p* = 0.449), eye disease (*p* = 0.304), tumor (*p* = 0.868), ASA classification (*p* = 0.474), anesthesia method (*p* = 0.693) and side of surgery (*p* = 0.462) between the two groups of patients.

**Table 1:** Baseline and clinical characteristics of ONFH patients in ARCO stage III (group A) and ARCO stage IV (group B)

Variables	Group A (*n* = 383)	Group B (*n* = 493)	*p* value
Age (years)	54.98 ± 12.89	55.99 ± 11.82	0.687
Sex (male), *n* (%)	243 (63.4%)	322 (65.4%)	0.567
BMI (kg/m^2^)	25.52 ± 3.89	25.48 ± 3.88	0.976
Place of residence (rural), *n* (%)	194 (50.6%)	194 (50.6%)	0.825
Tobacco smoker (yes), *n* (%)	49 (12.8%)	57 (11.6%)	0.579
Alcohol abuse (yes), *n* (%)	61 (15.9%)	67 (13.6%)	0.331
Hormone use (yes), *n* (%)	35 (9.1%)	56 (11.4%)	0.285
Hip injury experience (yes), *n* (%)	93 (24.3%)	104 (21.1%)	0.262
Anti-inflammatory painkiller (yes), *n* (%)	141 (36.8%)	189 (38.3%)	0.645
Hypertension (yes), *n* (%)	48 (12.5%)	73 (14.8%)	0.333
Diabetes (yes), *n* (%)	29 (7.6%)	34 (6.9%)	0.701
Anemia (yes), *n* (%)	20 (5.2%)	27 (5.5%)	0.868
Heart disease (yes), *n* (%)	18 (4.7%)	26 (5.3%)	0.700
Cerebrovascular disease (yes), *n* (%)	16 (4.2%)	31 (6.3%)	0.169
Pulmonary disease (yes), *n* (%)	4 (1.0%)	8 (1.6%)	0.465
Liver disease (yes), *n* (%)	19 (5.0%)	14 (2.8%)	0.102
Urinary system disease (yes), *n* (%)	8 (2.1%)	7 (1.4%)	0.449
Eye disease (yes), *n* (%)	11 (2.9%)	9 (1.8%)	0.304
Tumor (yes), *n* (%)	0 (0%)	1 (0.2%)	0.868
ASA classification (I), *n* (%)	126 (32.8%)	151 (30.6%)	0.474
Anesthesia method (general), *n* (%)	211 (55.1%)	265 (53.8%)	0.693
Surgery side (left), *n* (%)	173 (45.1%)	235 (47.6%)	0.462

### Postoperative outcomes

The details of intraoperative information and clinical outcomes are listed in Table 2. Among the intraoperative outcomes, the mean operation time in group A (66.71 ± 6.72 min) is shorter than that in group B (71.59 ± 8.24 min), with a significant difference between the two groups (*p* < 0.01). Group A also exhibited markedly less blood loss (239.92 ± 54.41 ml vs 265.35 ± 47.76 ml, *p* < 0.01) than group B, while group B patients had more one-year readmissions (*p* = 0.026) and one-year complications (*p* = 0.040). We also found no significant differences between the two groups in intraoperative transfusion (*p* = 0.088), time for ambulation (*p* = 0.466), LOS (*p* = 0.058), one-year mortality (*p* = 0.538), three-year complications (*p* = 0.169), five-year complications (*p* = 0.206), and overall revision (*p* = 0.102).

**Table 2:** Comparison of ARCO stage III (group A) and ARCO stage IV (group B) intraoperative information and clinical outcomes

Variables	Group A (*n* = 383)	Group B (*n* = 493)	*p* value
Intraoperative information			
Operation time (min)	66.71 ± 6.72	71.59 ± 8.24	< 0.01*
Blood loss (ml)	239.92 ± 54.41	265.35 ± 47.76	< 0.01*
Intraoperative transfusion, *n* (%)	25 (6.5%)	48 (9.7%)	0.088
Clinical outcomes			
Time for ambulation (days)	1.41 ± 0.66	1.44 ± 0.69	0.466
LOS (days)	14.61 ± 4.72	15.38 ± 5.08	0.058
One-year mortality, *n* (%)	4 (1.0%)	6 (1.2%)	0.538
One-year Readmissions, *n* (%)	11 (2.9%)	30 (6.1%)	0.026*
Complications, *n* (%)			
One-year, *n* (%)	19 (5.0%)	42 (8.5%)	0.040*
Three-year, *n* (%)	32 (8.4%)	55 (11.2%)	0.169
Five-year, *n* (%)	37 (9.7%)	61 (12.4%)	0.206
Overall revision, *n* (%)	14 (9.7%)	30 (12.4%)	0.102

*Bold: *p* < 0.05 indicates a significant difference

### Complication

**Fig. 2:**
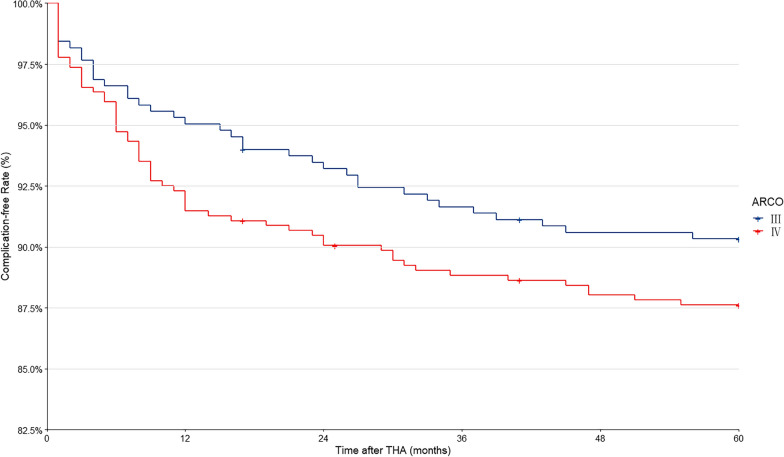
Complication-free rate between ARCO stage III (group A) and ARCO stage IV (group B) patients after THA during the five-year follow-up

As shown in detail in Fig. 2, 60 months complication-free rate was significantly higher for patients in ARCO stage III compared with ARCO stage IV. Table 3 summarises and compares all surgical complications after THA over a five-year period between the two groups. Local complications affected 23 (62.2%) hips in group A and 43 (70.5%) hips in group B. Systemic complications affected 14 (37.8%) patients in group A and 18 (29.5%) patients in group B. Of all causes, prosthesis dislocation appears to be higher in group B (4.1%) than in group A (1.6%), and their incidences are statistically different (*p* = 0.031). No significant differences between the two groups were noted in terms of other complications, such as pain, superficial infection and haematoma, etc.

**Table 3:** Total complications after THA in five years between ARCO stage III (group A) and ARCO stage IV (group B)

Surgical complication	Group A (*n* = 383)	Group B (*n* = 493)	*p* value
**Local complications**	**23 (62.2%)**	**44 (70.5%)**	
Pain	4 (10.8%)	3 (4.9%)	0.737
Superficial infection	4 (10.8%)	5 (8.2%)	1.000
Deep infection	3 (8.1%)	6 (9.8%)	0.769
Hematoma	1 (2.7%)	1 (1.6%)	1.000
Fracture	4 (10.8%)	5 (8.2%)	1.000
Dislocation	6 (16.2%)	20 (32.8%)	0.031*
**Systemic complications**	**14 (37.8%)**	**17 (29.5%)**	
Anemia	2 (5.4%)	5 (8.2%)	0.668
Deep venous thrombosis	4 (10.8%)	7 (11.5%)	0.850
Pulmonary complications	1 (2.7%)	1 (1.6%)	1.000
Gastrointestinal complications	1 (2.7%)	0 (0%)	0.437
Genitourinary complications	1 (2.7%)	1 (1.6%)	1.000
Neurological complications	3 (8.1%)	2 (3.3%)	0.777
Cardiovascular complication	3 (8.1%)	5 (8.2%)	1.000
**Total**	**37 (100%)**	**61 (100%)**	

*Bold: *p* < 0.05 indicates a significant difference

### Clinical scores

The HHS increased from 62.00 ± 5.22 to 91.96 ± 5.12, and the VAS score decreased from 6.79 ± 1.54 to 1.02 ± 0.09 in the stage III group. The HHS increased from 60.09 ± 5.12 to 90.17 ± 5.20, and the VAS score decreased from 6.93 ± 1.59 to 1.29 ± 0.07 in the stage IV group. THA provided significant hip function restoration and pain mitigation for patients with stage III and IV ONFH, as shown by the elevated HHS and reduced VAS scores. At the same time, patients in Group A had significantly higher postoperative HHS than those in Group B (91.96 ± 5.12 vs. 90.17 ± 5.20, *p* < 0.01) (Table 4).

**Table 4:** Comparison of ARCO stage III (group A) and ARCO stage IV (group B) preoperative and postoperative clinical scores

Variables	Group A (*n* = 383)	Group B (*n* = 493)	*p* value
Preoperatively			
VAS scores	6.79 ± 1.54	6.93 ± 1.59	0.064
HSS	62.00 ± 5.22	60.09 ± 5.12	< 0.01*
Postoperatively			
VAS scores	1.02 ± 0.65	1.29 ± 0.55	0.070
HSS	91.96 ± 5.12	90.17 ± 5.20	< 0.01*

*Bold: *p* < 0.05 indicates a significant difference

## Discussion

This study compared THA outcomes in the treatment of ARCO stage III and stage IV ONFH patients. Our results revealed that compared to stage III patients, ARCO stage IV patients had longer operative times, more intraoperative bleeding, more one-year readmissions and complications, and worse hip function at the one year follow-up. In addition, ARCO stage IV patients seem more likely to suffer prosthesis dislocation.

ONFH patients undergoing THA in ARCO stage IV had longer operative time and more intraoperative bleeding according to our survey, which was similar to the study by Yang et al.. Compared to ARCO stage III, which shows only femoral head collapse, ARCO stage IV results in cartilage damage on the acetabular side, and ONFH progresses to secondary osteoarthritis [20, 21]. Due to the more severe hip deformity, including acetabular wear, adherent joint capsule and muscle atrophy, the longitudinal axis of the joint is severely shortened in ARCO stage IV patients. The surgeon must spend extra time relaxing the stiff muscles around the area, opening the sclerotic joint capsule, and cleaning up the deformed acetabulum and osteophyte hyperplasia [22]. In addition, more time is spent identifying the position of the acetabular cup and the direction of placement, and repairing and reconstructing the external rotator group [23]. The above procedures significantly increase surgical wound exposure time, anesthesia risk and intraoperative bleeding and they are unavoidable [34–36].

During the five-year following-up, ARCO stage IV patients always showed higher overall complication rates than ARCO III patients, especially in the first year. Some reasonable mechanisms can explain this result. Patients with severe ONFH have prolonged bed rest due to significant pain and cause muscular flaccidity [24]. Slow blood flow and uneven tone of the muscle groups increases the risk of medical conditions such as DVT and cardiovascular and cerebrovascular disease [25]. Long-term immobilisation can also lead to poor health conditions, such as reduced cardiopulmonary function and gastricism, which affect early prognosis. In addition, psychological factors from patients with refractory hip disease, such as pain catastrophizing, anxiety, and depression, have also been shown to influence function and prognosis after THA, including higher rates of poor prognosis and more surgical complications [26, 27]. For patients with ARCO stage IV, hospitals should notice their mental health and provide appropriate psychological counseling while actively optimizing patient physical conditions.

We connected the ARCO stage with complaints of ONFH patients after THA for the first time and confirmed that ARCO IV patients are more prone to prosthesis dislocation. Patients with ARCO IV suffer from acetabular deformity and abnormal structure due to aseptic inflammation of the hip joint and friction of the deformed femoral head, which makes it more difficult to sand the acetabulum and accurately place the acetabular component, which is essential for preventing prosthesis dislocation. The external rotator group is also affected by painful stimuli and prolonged braking, resulting in muscle imbalances and disuse atrophy that do not provide stable mechanical support for the prosthesis. In addition, patients with hip osteoarthritis was characterized by significant changed gait and impaired coordination and balance [28]. Unfortunate events, such as stumbles, falls and even car accidents, can lead to associated soft tissue injuries and even dislocations [33]. In such cases, rehabilitation education of ARCO stage IV patients focused on avoiding early full weight-bearing and elimination of movements leading to dislocation, carried out by an experienced surgeon, can reduce the risk.

The differences between HHS showed that ARCO stage III patients have better early hip joint function after THA than those patient in ARCO stage IV. In normal physiological state, hip abductors, ball and socket joints and peripheral ligaments move in concert to complete the load bearing work together. However, ARCO stage IV patients suffer from aseptic inflammation and fatty infiltration, which leads to muscle atrophy and weakness, and adhesions of the peripheral ligaments, eventually causing severe movement disorders on the affected side and even gradual loss of strength on the contralateral healthy side [32]. Although adhesive soft tissue and stiff muscle release are lightly released during the surgery, the weak muscles and soft tissue changes still limit early activity [29]. In a prospective study of 222 ONFH patients treated with THA, Fortin et al. also concluded that patients with the worst function and pain at the time of surgery had worse function 2 years after surgery, which was attributed to the timing of THA [30]. Hospitals need to provide more scientific rehabilitation programmes for ARCO stage IV patients, such as more active ankle pump exercise and straight leg raise exercise, and urge them to train to obtain better muscular strength and joint functions [31].

In this study, we conducted a long-term follow-up to identify the difference of distant outcomes after THA between the two groups. However, there are still some limitations that we cannot avoid. First of all, our study population was all from the same institution. Although, the single-center study design allows minimization of interobserver variability, but may have introduced selection bias. In addition, distant complications were mainly collected by telephone follow-up, which means that accuracy greatly relies on patient self-report and knowledge of their medical conditions. Finally, all THAs in this study were performed using the posterolateral approach, which means that our findings may not be applicable to other surgical approaches. Fortunately, the posterolateral approach is widely accepted and used surgical approach, and we believe that our results are reliable for the vast majority of patients.

## Conclusion

Although ARCO stage IV patients in our study cohorts are likely to suffer more one-year complications, no significant difference was observed at long-term follow-up. Increasing awareness of preventing early prosthesis dislocation may help improve the prognosis of final-stage ONFH patients.

## Data Availability

All the data used are available from the corresponding author on motivated requests.
